# The effect of umbilical cord coiling ındex measured in antenatal period on pregnancy results

**DOI:** 10.1007/s00404-026-08342-1

**Published:** 2026-02-04

**Authors:** Murat Cengiz, Ercan Yilmaz

**Affiliations:** 1 Department of Obstetrics and Gynecology, Faculty of Medicine, Hacettepe University, 06230 Ankara, Turkey; 2 Department of Obstetrics and Gynecology, Faculty of Medicine, Inonu University, Malatya, Turkey

**Keywords:** Umblical cord, Ultrasonography, Prenatal, Primigravida, Placenta, Screening, Prenatal

## Abstract

**Purpose:**

To evaluate the association between ultrasonographically measured umbilical coiling index (UCI) at 18–24 weeks of gestation and adverse perinatal outcomes in primigravid pregnancies.

**Methods:**

This prospective study included 461 primigravid women with singleton pregnancies. UCI was measured at 3 cord segments and classified as hypocoiled (< 0.20), normocoiled (0.20–0.40), or hypercoiled (> 0.40) using percentile distribution and ROC-derived thresholds. Maternal characteristics, delivery outcomes, fetal well-being, placental measurements, cord blood gas values, and neonatal outcomes were compared using Kruskal–Wallis, Mann–Whitney U, and chi-square tests (p < 0.05 was significant).

**Results:**

Of the 461 patients, 72 (15.6%) were hypocoiled, 244 (52.9%) normocoiled, and 145 (31.5%) hypercoiled. No significant differences were found in maternal age, BMI, gestational age at delivery, hypertension, diabetes, or placental abruption. Birth weight was lowest in the hypocoiled group (p < 0.001). Umbilical artery pH was significantly lower in the hypercoiled group (p < 0.001). Both hypo and hypercoiled groups showed significantly reduced placental weight/thickness (p < 0.001) and higher rates of non-reassuring non-stress tests (34.7 and 28.3% vs. 9.0%, p < 0.001). Meconium-stained amniotic fluid (p = 0.003), oligohydramnios (p < 0.001), and intrauterine growth restriction (p < 0.001) were more common in abnormal coiling groups. Five-minute Apgar scores were significantly lower in both abnormal groups (p < 0.001). No association was found with fetal death (p = 0.575).

**Conclusion:**

Both decreased and excessive umbilical cord coiling in the second trimester are associated with impaired fetal growth and adverse perinatal outcomes. Routine second-trimester UCI assessment may help identify high-risk pregnancies.

## What does this study add to the clinical work?

**Table:** 

This study demonstrates that both hypo- and hypercoiling of the umbilical cord identified in the second trimester are associated with adverse perinatal outcomes, including impaired fetal growth and non-reassuring intrapartum fetal status. Routine antenatal assessment of the umbilical coiling index may help identify pregnancies at increased risk and improve antenatal surveillance strategies.	

## Introduction

The umbilical cord, which connects the fetus to the placenta and serves as a conduit for the exchange of essential nutrients, gases, and fluids between the mother and fetus during intrauterine life, normally consists of 2 arteries and one vein [1]. Pathologic alterations involving the placenta or the umbilical cord may adversely affect fetal growth and development [2]. In recent years, numerous studies have explored the association between the degree of umbilical cord coiling and various maternal and fetal complications [3–6].

The rudimentary umbilical cord develops between the 4th and 8th weeks of gestation and is composed of amniotic tissue derived from the body stalk, the omphalomesenteric duct, and the umbilical coelom [7]. Blood flow within the umbilical cord begins by the 5th week of gestation [8]. Various theories have been proposed to explain the spiral configuration of the umbilical cord, including the hypothesis that it results from fetal movements [6, 9]. The direction of the spiral is usually counterclockwise, and its helical structure can be visualized sonographically as early as the first trimester, becoming clearly apparent by the 9th week of gestation. The total number of vascular coils averages around 40 but may range widely, even up to 380 in some cases. The number of coils can be assessed more reliably during the first and second trimesters, as it shows minimal increase during the third trimester [10]. Therefore, the length of the umbilical cord is primarily determined by the distance between successive coils rather than by the total number of coils.

The coiled configuration of the umbilical cord was first described by Berengarius in 1521, as cited by Edmonds [11]. The degree of umbilical coiling can be quantified using the umbilical coiling ındex (UCI), defined as the number of complete vascular coils per unit length of the umbilical cord. Sonographically, the index may also be estimated by dividing the distance required for a single complete spiral of the umbilical artery around the umbilical vein [12].

The second trimester is generally considered the most suitable period for ultrasonographic assessment. During this stage, the fetal structures are optimally visualized within the amniotic fluid, and even the boundaries between the umbilical arteries and vein can be clearly delineated [13].

Abnormalities in umbilical cord coiling have been associated with adverse perinatal outcomes, including intrauterine growth restriction, fetal distress, preterm birth, and stillbirth [1, 14, 15]. However, given the limited number of prospective studies on this topic, we conducted a prospective study to evaluate the effect of the umbilical coiling index (UCI), measured ultrasonographically between 18 and 24 weeks of gestation in primigravid pregnancies, on fetal outcomes.

## Materials and methods

### Study population

Between January 2018 and August 2019, a total of 461 primigravid women with singleton pregnancies between 18 and 24 weeks of gestation who presented to the department of obstetrics and gynecology at Inonu University Turgut Ozal Medical Center were prospectively enrolled in the study. Umbilical coiling index (UCI) measurements were performed by a single experienced clinician to ensure consistency. For each participant, the following parameters were prospectively recorded in a standardized manner: mode of delivery, gestational age at delivery, maternal complications developing during pregnancy, fetal conditions (such as intrauterine growth restriction), cord blood pH, birth weight, neonatal sex, presence of meconium-stained amniotic fluid, placental weight, and placental thickness measured in the horizontal plane. The 5 min Apgar score was also documented for each neonate. Oligohydramnios was defined as a maximum vertical pocket (MVP) < 2 cm.

Only primigravid women with singleton pregnancies between 18 and 24 weeks of gestation were included in the study. All participants had viable fetuses without any structural anomaly on the second-trimester ultrasound, and adequate visualization of the umbilical cord was required to ensure accurate UCI measurement. Women were enrolled only after providing written informed consent.

Patients were excluded if they had multiple gestations (twin or higher-order pregnancies), known fetal structural or chromosomal abnormalities, or pregestational maternal diseases such as type 1 or type 2 diabetes mellitus, chronic hypertension, renal disease, or autoimmune disorders. Pregnancies complicated by placental abnormalities diagnosed before or during the study, or cases in which the umbilical cord could not be adequately visualized, were also excluded. Additionally, women who developed major obstetric complications before UCI assessment or those with incomplete maternal or neonatal follow-up data were not included in the final analysis.

### Umbilical coiling index measurement

**Fig. 1:**
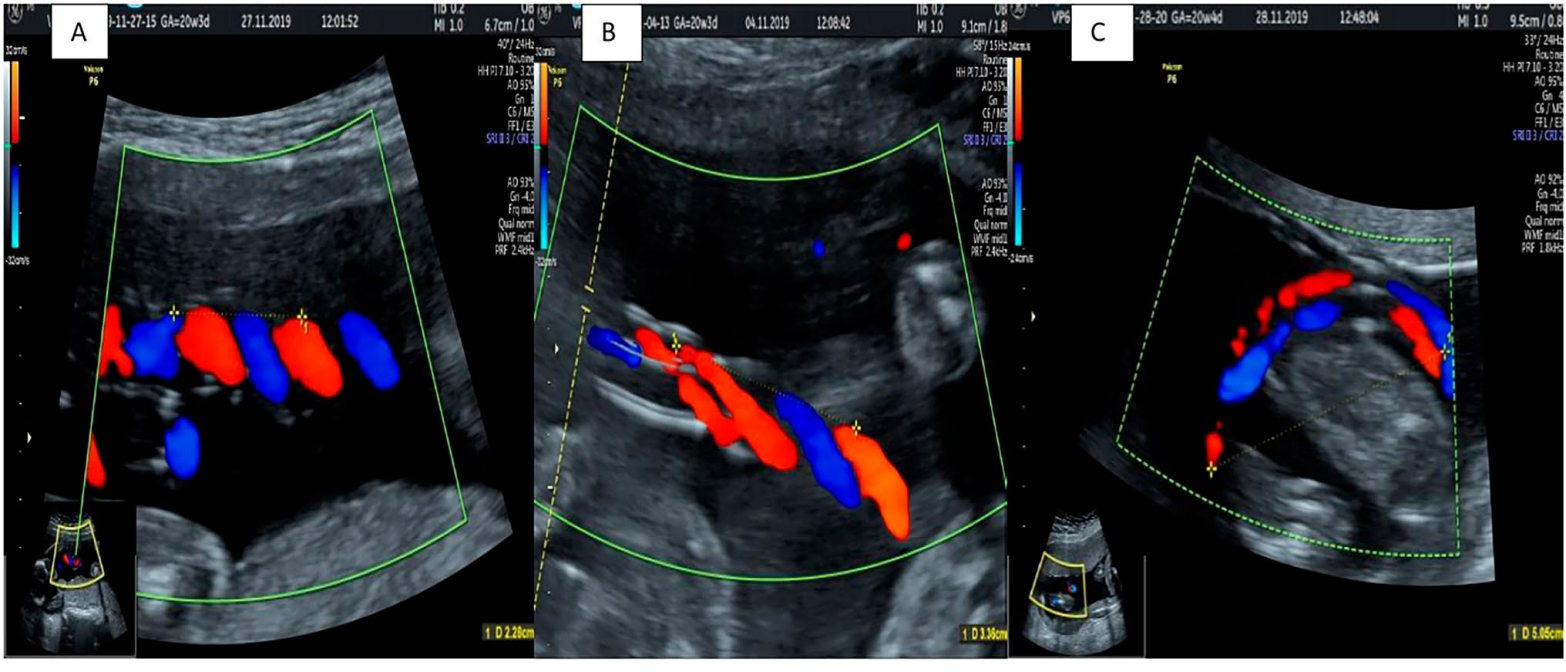
Ultrasonographic assessment of umbilical cord coiling patterns

The umbilical coiling index (UCI) was calculated sonographically by dividing one by the distance (in centimeters) required for the umbilical artery to complete a single spiral turn around the umbilical vein (UCI = 1/distance in cm) (Fig. 1). Measurements were obtained using a Voluson E6 ultrasound system (GE Healthcare, Milwaukee, WI, USA) equipped with a 4C-D 2–5 MHz convex transducer. Coiling measurements were taken at 3 distinct cord segments: near the placental insertion, at the middle portion, and near the fetal insertion; the mean of these 3 measurements was used as the final UCI value.

The umbilical coiling index (UCI) was categorized using both percentile distribution and receiver operating characteristic (ROC) curve analysis. Percentile values were used to define hypocoiled and hypercoiled cords, respectively, while ROC analysis was employed to determine the optimal cut-off values for predicting adverse perinatal outcomes. Based on these analyses, the final categorization was established as hypocoiled (< 0.2), normocoiled (0.2–0.4), and hypercoiled (> 0.4).

### Management of delivery

The mode of delivery was determined based on obstetric indications and clinical follow-up. As all participants were primigravidas, cesarean section was performed when clinically indicated, while others underwent spontaneous vaginal delivery following active management of labor.

### Placental measurements

Following delivery, the placenta was weighed using a precision scale. After being placed in the anatomical position on a flat surface, its thickness was measured in the horizontal plane. All measurements were recorded using the patient’s study identification number.

### Umbilical cord blood gas analysis

Immediately after delivery, the umbilical cord was clamped, and a sample was aspirated into a heparinized syringe for blood gas analysis. All samples were analyzed promptly using the same blood gas analyzer available at the hospital to ensure consistency.

### Statistical analysis

Statistical analyses were performed using IBM SPSS Statistics for Windows, Version 22.0 (IBM Corp., Armonk, NY, USA). The Kolmogorov–Smirnov test was used to assess the normality of continuous variables. Normally distributed variables were compared using the independent samples t test or one-way ANOVA, whereas non-normally distributed variables were analyzed with the Mann–Whitney U or Kruskal–Wallis tests. The chi-square test was used to evaluate associations between categorical variables. Cut-off values for the umbilical coiling index (UCI) were determined using both receiver operating characteristic (ROC) curve analysis and percentile distribution. A p value < 0.05 was considered statistically significant.

## Results

A total of 461 primigravid patients were included. The participants’ ages ranged from 17 to 41 years (mean 26.6 ± 4.18). Ultrasonographic examinations were performed between 18 and 24 gestational weeks (mean 20.4 ± 1.8). The gestational age at delivery ranged from 24 to 42 weeks (mean 38.2 ± 2.2) (Table 1).

**Table 1:** Maternal and obstetric characteristics (n = 461)

Variable	Minimum	Maximum	Mean ± SD
Maternal age (years)	17	41	26.6 ± 4.18
Gestational age at ultrasound (weeks)	18	24	20.4 ± 1.8
Gestational age at delivery (weeks)	24	42	38.2 ± 2.2
Maternal BMI (kg/m^2^)	17	42	24.8 ± 3.3
Placental weight (g)	175	1000	543.3 ± 87.9
Placental thickness (cm)	1	4	2.43 ± 0.34
Umbilical cord blood pH	7.00	7.44	7.31 ± 0.04

Neonatal birth weight ranged from 545 to 4700 g (mean 3130.6 ± 556.2 g). Maternal BMI ranged from 17 to 42 kg/m^2^ (mean 24.8 ± 3.3). Mean placental weight was 543.3 ± 87.9 g and placental thickness averaged 2.43 ± 0.34 cm. Umbilical cord arterial pH ranged from 7.00 to 7.44 (mean 7.31 ± 0.04). Among the newborns, 222 (48.2%) were male and 239 (51.8%) were female. A total of 167 (36.2%) patients delivered vaginally and 294 (63.8%) by cesarean section. Cesarean delivery rates differed significantly among groups (p = 0.029), highest in the hypercoiled group (Table 2).

**Table 2:** Maternal, obstetric, and neonatal outcomes by UCI category

Variable	Hypocoiled	Normocoiled	Hypercoiled	p value
N	72	244	145	–
Maternal age (years)	26.0 (median)	26.0	26.0	0.724
Gestational age at delivery (weeks)	~ 38	~ 38	~ 38	NS
Maternal BMI (kg/m^2^)	25.0 ± 3.8	25.0 ± 3.46	24.6 ± 2.88	0.650
GDM (%)	2.8%	6.6%	3.4%	0.252
Gestational hypertension (%)	2.8%	2.0%	2.8%	0.922
Cesarean delivery (%)	56.9%	60.7%	72.4%	0.029
Oligohydramnios (%)	29.2%	5.3%	9.7%	< 0.001
Meconium (%)	9.7%	3.3%	11.7%	0.003
Fetal death (%)	1.4%	0.8%	0%	0.575
Placental abruption (%)	1.4%	0.4%	1.4%	0.482
Non-reassuring NST (%)	34.7%	9.0%	28.3%	< 0.001
IUGR overall (%)	26.4%	2.4%	~ 9.0%	< 0.001
Late-onset IUGR ≥ 32 wk (%)	20.8%	1.6%	6.9%	< 0.001
Early-onset IUGR < 32 wk (%)	5.6%	0.8%	2.1%	< 0.001

*UCI* Umbilical coiling Index, *BMI* Body mass index, *GDM* Gestational diabetes mellitus, *NST* Non-stress test, IUGR Intrauterine growth restriction

ROC coordinate analysis demonstrated that UCI values around 0.20 provided high sensitivity (0.79–0.84), supporting the choice of < 0.20 as a clinically meaningful hypocoiling cut-off. Conversely, UCI values exceeding 0.40 were associated with lower sensitivity but relatively higher specificity (> 70%), indicating a distinct high-coiling phenotype. Given the modest overall discriminative capacity of UCI on the ROC curve, percentile-derived thresholds (5th percentile = 0.19 and 75th percentile = 0.41) were used, consistent with the ROC characteristics and published literatüre.

Maternal age did not differ among hypocoiled, normocoiled, and hypercoiled groups (Kruskal–Wallis χ^2^ = 0.647, p = 0.724). Gestational age at delivery was also similar across groups (≈38 weeks). There was no significant difference in maternal BMI (p = 0.650), gestational hypertension (p = 0.922), GDM (p = 0.252), placental abruption (p = 0.482), or fetal death (p = 0.575) (Table 2).

Oligohydramnios was more frequent in the hypercoiled group (9.7%) and hypocoiled group (29.2%) compared with normocoiled (5.3%) (p < 0.001). Meconium-stained amniotic fluid was significantly more common in both hypocoiled (9.7%) and hypercoiled (11.7%) groups compared with the normocoiled group (3.3%) (p = 0.003) (Table 2).

A significant association was found between UCI category and the presence and timing of intrauterine growth restriction (IUGR) (Pearson χ^2^ = 42.566, p < 0.001). Late-onset IUGR (≥ 32 weeks) was markedly more frequent in the hypocoiled group (20.8%) compared with the normocoiled (1.6%) and hypercoiled groups (6.9%). Early-onset IUGR (< 32 weeks) also occurred more commonly in the hypocoiled group (5.6%) than in the normocoiled (0.8%) and hypercoiled groups (2.1%). The normocoiled group had the lowest overall IUGR incidence (2.4%), whereas both hypocoiling and hypercoiling were associated with higher IUGR rates, particularly late-onset disease (Table 2).

The relationship between UCI categories and intrapartum fetal status was also evaluated. A significant association was found between UCI category and the presence of non-reassuring NST findings (χ^2^ = 35.345, p < 0.001). Non-reassuring NST findings were most frequent in the hypocoiled group (34.7%), followed by the hypercoiled group (28.3%), whereas the normocoiled group showed the lowest rate (9.0%). Conversely, the proportion of fetuses with reassuring NST tracings was highest in the normocoiled group (91.0%) and lowest in the hypocoiled group (65.3%). These results indicate that both hypocoiling and hypercoiling are associated with a higher likelihood of non-reassuring intrapartum fetal status, while normocoiling appears to be the most favorable pattern (Table 2).

Birth weight differed significantly among hypocoiled, normocoiled, and hypercoiled groups (Kruskal–Wallis χ^2^ = 40.684, p < 0.001). The hypocoiled group had the lowest birth weight ranks, while the normocoiled group had the highest. When neonatal birth weights were evaluated, the mean birth weight was 2855.6 ± 571 g in the hypocoiled group, 3237.0 ± 528 g in the normocoiled group, and 3087.1 ± 545 g in the hypercoiled group. Pairwise Mann–Whitney U analyses revealed that birth weight significantly differed between all UCI categories. Neonates in the hypocoiled group had significantly lower birth weights compared with both the normocoiled (p < 0.001) and hypercoiled groups (p < 0.001). Additionally, birth weight was higher in the normocoiled group than in the hypercoiled group (p = 0.007). Overall, the hypocoiled group consistently demonstrated the lowest birth weight values, indicating a strong association between reduced coiling and fetal growth. (Table 3).

**Table 3:** Neonatal outcomes by UCI category

Neonatal Outcome	Hypocoiled	Normocoiled	Hypercoiled	p value
Birth weight (g)	2855.6 ± 571	3237.0 ± 528	3087.1 ± 545	< 0.001
Cord pH	7.30 ± 0.02	7.32 ± 0.03	7.28 ± 0.04	< 0.001
Apgar < 5 (%)	4.2%	0.8%	2.8%	< 0.001
Apgar 5–7 (%)	50.0%	6.1%	33.8%	< 0.001
Apgar ≥ 7 (%)	45.8%	93.0%	63.4%	< 0.001
Placental weight (g)	500.5 ± 71.8	559.3 ± 78.4	537.7 ± 101.8	< 0.001
Placental thickness (cm)	2.24 ± 0.32	2.51 ± 0.33	2.39 ± 0.33	< 0.001

*UCI* Umbilical coiling Index, *pH* Umbilical cord arterial pH

Regarding umbilical cord blood gas pH, the mean pH was 7.30 ± 0.02 in the hypocoiled group, 7.32 ± 0.03 in the normocoiled group, and 7.28 ± 0.04 in the hypercoiled group. Umbilical cord arterial pH values differed significantly among the 3 UCI groups (Kruskal–Wallis χ^2^ = 171.934, p < 0.001). Post-hoc pairwise Mann–Whitney U tests demonstrated that the normocoiled group had significantly higher pH values than both the hypocoiled (p < 0.001) and hypercoiled groups (p < 0.001). Additionally, pH values in the hypocoiled group were significantly higher than those observed in the hypercoiled group (p < 0.001). Overall, the hypercoiled group exhibited the lowest pH levels, whereas the normocoiled group had the highest (Table 3).

Placental weight was significantly lower in the hypocoiled group (500.5 ± 71.8 g) compared with the normocoiled (559.3 ± 78.4 g) and hypercoiled groups (537.7 ± 101.8 g) (p < 0.001). Placental thickness also differed significantly (p < 0.001), with both hypocoiled and hypercoiled cords showing reduced thickness compared with normocoiled cords (Table 3).

Apgar scores at 5 min were also compared among the groups. Scores were categorized as follows: < 5 (code 1), 5–7 (code 2), and ≥ 7 (code 3). A significant association was observed between UCI category and 5 min Apgar score ( χ^2^ = 88.297, p < 0.001). Low and intermediate Apgar scores were more frequent in the hypocoiled group (4.2 and 50.0%, respectively), whereas the normocoiled group demonstrated the highest proportion of neonates with normal Apgar scores (93.0%). In contrast, the hypercoiled group showed a higher proportion of intermediate Apgar scores compared with the normocoiled group (33.8% vs. 6.1%). These findings indicate that both reduced and excessive coiling are associated with impaired immediate neonatal condition (Table 3).

## Discussion

The umbilical cord plays a fundamental role in sustaining fetal nutrition within the womb, and its helical structure is essential to this function. Although several theories have been proposed to explain the origin of cord coiling, no definitive consensus has been reached. The most widely accepted hypothesis attributes coiling to a genetically determined interaction between fetal movement and vascular growth patterns [16, 17]. Structural studies have shown that the umbilical artery consists of 4 distinct layers—an inner circular layer, a longitudinal layer, a main circular muscle layer, and a small circular muscle layer—which together contribute to the cord’s natural helical configuration [18]. This spiral architecture provides mechanical strength and flexibility, helping to maintain uninterrupted blood flow despite external forces [19]. Numerous studies have shown that extremes of umbilical cord coiling—either excessive or reduced—are associated with adverse prenatal and neonatal outcomes [19–21].

In this study, we aimed to identify potentially predictable conditions during routine second-trimester evaluations in primigravid women. Restricting the study population to primigravid patients allowed for a more precise assessment of the relationship between umbilical coiling patterns, the frequency of operative deliveries, and unfavorable fetal conditions.

The first study investigating the implications of cord coiling was conducted by Strong et al. [14], who reported that intrauterine fetal demise, preterm birth, operative delivery for fetal distress, and meconium-stained amniotic fluid were more frequent in non-normocoiled cords. Our findings were broadly consistent with these observations: both hypo and hypercoiled cords were associated with several adverse pregnancy outcomes compared with the normocoiled group. Notably, the rate of non-reassuring fetal status during labor was significantly higher in both abnormal coiling groups (p < 0.001). While Rana et al. [1] reported increased fetal distress only in hypocoiled cords. Predanic et al. [22] found no significant relationship between coiling and fetal distress. Ezimokhai et al. [3], however, reported increased operative delivery due to fetal distress in the hypercoiled group.

Several mechanistic explanations have been proposed. De Laat et al. [23] suggested that hypercoiling increases arterial pressure, compromising perfusion in terminal capillaries. Ernst et al. [24] noted a higher risk of vascular obstruction in hypercoiled cords. Based on our findings, we also hypothesize that hypocoiling may impair the continuity of end-systolic pressure transmission within the umbilical arterial wall, reducing blood flow stability. However, definitive mechanistic conclusions require Doppler velocimetry studies.

In our study, both hypocoiled and hypercoiled cords were associated with lower umbilical arterial pH values compared with normocoiled cords, suggesting an increased risk of fetal acidemia. Although severe neonatal complications occur when arterial pH falls below 7.0 [25, 26], and pH values below 7.2 have been linked to neonatal seizures [27], blood gas values in our cohort did not reach pathologic thresholds. Thus, while abnormal coiling was associated with statistically lower pH values, these differences did not translate into clinically severe neonatal acidemia.

Placental morphology also appeared to parallel abnormal coiling patterns. Adequate placental surface area is crucial for fetal oxygenation during labor, and reductions in placental mass may affect blood gas values. In our study, placental weight and thickness were significantly lower in hypo and hypercoiled cords, paralleling reductions in birth weight. This aligns with previous research showing a strong correlation between placental and fetal weigh [28]. To date, no studies have directly examined the association between placental dimensions and coiling index, and future investigations incorporating histopathological analyses may clarify whether abnormal coiling reflects underlying placental developmental abnormalities.

Neonatal outcomes showed comparable trends, with 5 min Apgar scores significantly lower in both hypo and hypercoiled groups. This finding aligns with the observations reported by Gupta et al. [29] and De Laat et al. [30, 31]. Similarly, Tran et al. [32] also demonstrated reduced 5 min Apgar scores in abnormal coiling cases. Meconium-stained amniotic fluid was also significantly more frequent in both abnormal coiling groups, in line with observations by Patil et al., Sharma et al., [12, 33, 34] and a meta-analysis. These findings collectively suggest that deviations from normal coiling may predispose the fetus to intrapartum stress.

Fetal growth parameters further supported this interpretation. Infants in both the hypocoiling and hypercoiling groups had significantly lower birth weights, consistent with prior studies and meta-analytic data [30, 32, 34]. IUGR was also more prevalent in both abnormal coiling groups in our cohort. Some earlier studies have reported increased IUGR primarily in hypercoiled cords [21, 33], while others found the strongest association in hypocoiled cords [35, 36]. Our study is the first, to our knowledge, to separately evaluate early and late-onset IUGR in relation to coiling. Both early (< 32 weeks) and late (≥ 32 weeks) IUGR followed a similar pattern—highest in hypocoiled cords—indicating that reduced coiling may be an especially strong marker of impaired fetoplacental function.

Oligohydramnios also showed a strong association with abnormal coiling. It was most frequent in the hypocoiled group but also significantly elevated in hypercoiled cords. Prior studies have demonstrated similar patterns, including a higher rate of oligohydramnios in both abnormal coiling groups [32, 35, 37]. Edmonds proposed that low amniotic fluid volume may restrict fetal movement, contributing to reduced coiling [11], which is compatible with our hypocoiling findings. However, this theory does not explain the increased oligohydramnios in hypercoiling. Reduced syncytiocapillary windows—observed histologically in hypercoiled placentas—may reduce diffusional exchange at the maternal–fetal interface, providing a plausible mechanism for oligohydramnios in excessive coiling [23].

The relationship between coiling and fetal death remains inconclusive. Some studies have reported increased fetal death in hypo or hypercoiled cords [21, 30], whereas others have found associations only in non-coiled cords [3], or in both extremes [34]. In our study, there was no significant association between coiling patterns and fetal death. Differences among studies may relate to methodological variation—particularly assessments performed after fetal demise, which may introduce bias.

Gestational age at delivery was not associated with coiling patterns in our study, consistent with results by Ezimokhai et al. [3]. Other studies have reported increased preterm birth in either hypercoiled [1], hypocoiled [37] or both groups [12, 30]. These inconsistencies likely reflect the multifactorial etiology of preterm labor, making it difficult to isolate the independent contribution of cord coiling.

Metabolic factors showed no clear association with coiling. We found no significant relationship between GDM or maternal BMI and coiling patterns, consistent with the heterogeneous and contradictory nature of prior findings [3, 37].

Finally, hypertensive disorders of pregnancy—including gestational hypertension and early- and late-onset preeclampsia—showed no significant association with coiling in our cohort. While some studies have suggested increased hypertensive disorders in abnormal coiling [35, 37], others, including major meta-analytic data, have found no such relationship [34, 38]. Similarly, placental abruption was not associated with coiling in our study, matching the findings of De Laat et al. [38], although some authors have reported an increased incidence of abruption in hypocoiled cords [37]. Given the multifactorial origins of hypertensive disorders and placental abruption—including inadequate trophoblastic invasion, inflammation, and infection [39, 40], it is unlikely that umbilical coiling alone explains these conditions.

## Conclusion

In this prospective cohort of 461 primigravid pregnancies assessed at 18–24 weeks, both reduced and excessive umbilical cord coiling were associated with adverse perinatal findings compared with normocoiling. Hypocoiling and hypercoiling correlated with lower birth weight, lower umbilical arterial pH, higher rates of meconium-stained amniotic fluid, oligohydramnios, and non-reassuring intrapartum fetal status, while normocoiling was associated with the most favorable outcomes. The hypocoiled group consistently showed the strongest signal for impaired fetal growth, including higher rates of IUGR—both early- and late-onset—whereas maternal age, BMI, GDM, hypertensive disorders, placental abruption, and fetal death did not differ significantly by UCI category.

While these findings support incorporating UCI into routine second-trimester risk stratification in primigravid patients, causality cannot be inferred and clinically severe neonatal acidemia was uncommon. Future studies should validate standardized UCI cut-offs across gestational ages, integrate Doppler velocimetry and placental histopathology, and evaluate whether UCI-guided surveillance or delivery planning improves neonatal outcomes.

## Data Availability

The datasets generated and/or analyzed during the current study are available from the corresponding author on reasonable request.

## Declarations

### Permission to reproduce material

Not applicable (no third-party material was reproduced).
